# Retraction Note: MicroRNA-320a acts as a tumor suppressor by targeting BCR/ABL oncogene in chronic myeloid leukemia

**DOI:** 10.1038/s41598-023-30736-3

**Published:** 2023-03-02

**Authors:** Zhu Xishan, Lin Ziying, Du Jing, Liu Gang

**Affiliations:** 1 Clinical Research Center, Affiliated Hospital of Guangdong Medical College, Dongguang, 0086-027-7398722 China; 2 Department of Urology, Weifang Traditional Chinese Medicine Hospital, Weifang, 0086-0536-8300338 China

Retraction of: *Scientific Reports* 10.1038/srep12460, published online 31 July 2015

The Editors have retracted this article.

After publication the following concerns were raised:

- Figure 6B (panel Fibronectin) appears to overlap with Figure 3B (panel PPM1D vs COLO-320 cells) of^1^.
- Figure 6B (panel Actin) appears to overlap with Figure 3B (panel β-actin vs COLO-320 cells) of^1^.
- Significant portions of text in this article overlap with text in^2^

The Editors therefore no longer have confidence in the results and conclusions presented.

The Editors were not able to obtain current email addresses for Zhu Xishan, Lin Ziying, Du Jing and Liu Gang.
